# The Experience of Family Members in Long-Term Care Facilities During the SARS COV2 (COVID-19) Pandemic

**DOI:** 10.1093/geroni/igab046.1248

**Published:** 2021-12-17

**Authors:** Raeann LeBlanc, Hyeyoung Park

**Affiliations:** 1 University of Massachusetts, Amherst, Royalston, Massachusetts, United States; 2 University of Massachusetts Amherst, Amherst, Massachusetts, United States

## Abstract

In-person visits are one form of care support offered by family members for residents in a Long-term Care Facility (LTCF). Family member visitation may extend to close social relationships with the residential care staff, which can be important in managing care. The long-term care population has been significantly impacted by a high number of SARS-COV2 (Covid-19) cases in morbidity and mortality but, in-person visits were limited due the public health concern. This study aimed to describe the experience of family members of persons in LTCFs during the Covid-19 pandemic. We used an online survey of 34 questions. Forty-six family members were recruited through online caregiver support platforms, and 22 completed the survey. Average participant age was 57. Majority were female with high-moderate (M=3.48) Kessler psychological distress scores. Participants reported less frequent communication with their family members in LTCFs. Difficult to reach nursing staff, who were the primary contact, was a concern. Their preferred means of communication was the telephone followed by window visits; residents preference remained for in-person visits followed by telephone. Participants described a decrease in relationship closeness with staff and a decrease in confidence in the quality of care. These results, limited by sample size, offer a beginning insight into the importance of communication between the family member and nursing staff, including the contact frequency. Structural disparities such as LTCF nursing staff levels may partly explain these deficits in supporting families during the Covid-19 pandemic. Opportunities to support family members remain a needed focus of long-term care reforms.

